# Frequent Ramen consumption and increased mortality risk in specific subgroups: A Yamagata cohort study

**DOI:** 10.1016/j.jnha.2025.100643

**Published:** 2025-08-01

**Authors:** Miho Suzuki, Natsuko Suzuki, Ri Sho, Masayoshi Souri, Tsuneo Konta

**Affiliations:** a Department of Health and Nutrition, Yamagata Prefectural Yonezawa University of Nutrition Sciences, Japan; b Department of Public Health and Hygiene, Yamagata University School of Medical Science, Japan

**Keywords:** Ramen noodles, Mortality, Salt intake, Yamagata cohort study

## Abstract

**Background:**

The frequent intake of ramen noodles may increase the risk of mortality from cardiovascular diseases and cancer. This study examined the association between ramen noodle intake frequency and mortality in Japan.

**Methods:**

This study included 6,725 participants aged ≥40 years from a questionnaire survey of the Yamagata cohort study. The average frequency of noodle intake was classified into four groups (<1/month, 1–3 times/month, 1–2 times/week, and ≥3 times/week). Cox proportional hazard analysis was performed to clarify the association between intake frequency and mortality.

**Results:**

The prevalence of ramen intake frequency was 18.9%, 46.7%, 27.0%, and 7.4% for <1/month, 1–3 times/month, 1–2 times/week, and ≥3 times/week, respectively. Participants with frequent ramen intake had higher body mass index values and a higher proportion of younger participants, men, smoking, alcohol consumption, diabetes, and hypertension. In multivariate Cox proportional analysis adjusted for various background factors, the “≥3 times/week” group showed a non-significantly increased risk for mortality (hazard ratio; 1.52, 95% confidence interval; 0.84–2.75), compared to the “1–2 times/week” group. In the subgroup analysis, the “≥3 times/week” group showed an increased risk of mortality in men, aged <70 years and those who consumed ≥50% of the noodle soup and alcohol.

**Conclusions:**

This study showed that Japanese community residents frequently consume ramen noodles and high intake is associated with various comorbidities. Frequent intake of ramen noodles might be associated with mortality risk in men, aged <70 years and those who consumed ≥50% of the noodle soup and alcohol.

## Highlights

• High intake of ramen noodles is associated with various comorbidities such as diabetes and hypertension.
• Frequent consumption of ramen may be associated with mortality risk in men under the age of 70.
• Mortality risk may also be higher among those who consume more than 50% of the noodle soup and those who consume alcohol.

## 1 Background

Ramen noodles, comprising wheat noodles and various ingredients, such as meat, vegetables, and seaweed, are among the most popular foods in Japan [1]. According to a household survey (2021–2023) by the Ministry of Internal Affairs and Communications, the amount of annual expenditure and quantity purchased per household in Japan is higher than for other Japanese noodles, such as soba and udon noodles [2]. Ramen noodles and their soup contain high salt content; therefore, frequent intake can lead to high amounts of sodium, which may increase the risk of salt-related diseases, such as stroke [3,4] and gastric cancer [5].

According to the Dietary Reference Intakes for Japanese (FY2020 version), the recommended salt intake is <7.5 g for men and <6.5 g for women [6]. However, the 2023 National Health and Nutrition Survey showed that the average salt intake was 9.8 g (10.7 g for men and 9.1 g for women), which is higher than the target value [7]. Excessive ramen intake may affect health status through excessive salt intake. A previous Japanese report showed that the number of ramen stores per population is correlated with death from stroke [8]. However, the association between ramen intake at an individual level and health outcomes has not yet been examined. This study examined the association between ramen intake frequency and mortality in the general Japanese population using information from the Yamagata Cohort Study Food Intake Frequency Questionnaire.

## 2 Methods

### 2.1 Study participants and procedures

Data from 6,746 individuals (2,361 men and 4,385 women) were used. After excluding 21 participants who died within the first year of follow-up, a total of 6,725 (2,349 men and 4,376 women) were analyzed. The self-administered questionnaire included information on personal background factors and frequency of ramen consumption. The questionnaire was distributed and collected by mail. The follow-up period was until December 2023. Information on total deaths was obtained from death certificates.

### 2.2 Survey items

#### 2.2.1 Personal characteristics and background factors

Information on personal characteristics (sex, age, height, weight, body mass index [BMI], smoking, alcohol consumption, and presence of hypertension, diabetes, and dyslipidaemia) were obtained through questionnaires and annual health examinations. Hypertension was defined as systolic blood pressure of ≥140 mmHg, diastolic blood pressure of ≥90 mmHg, or use of antihypertensive drugs. Diabetes mellitus was defined as fasting blood glucose ≥126 mg/dL, HbA1c ≥6.5%, or use of antidiabetic drugs. Dyslipidaemia was defined as triglycerides ≥150 mg/dL, low-density lipoprotein cholesterol ≥140 mg/dL, high-density lipoprotein cholesterol <40 mg/dL, or use of lipid-lowering drugs.

#### 2.2.2 Frequency of ramen noodle intake and amount of noodle soup consumed

In the question on the frequency of ramen intake on the survey form, the average frequency of ramen intake over the past year was asked in nine levels (<1/month, 1–3 times/month, 1–2 times/week, 3–4 times/week, 5–6 times/week, 1 time/day, 2–3 times/day, 4–6 times/day, and ≥7 times/day). Since the number of respondents in the groups of ≥5–6 times/week was small, they were divided into four groups (<1/month, 1–3 times/month, 1–2 times/week, and ≥3 times/week). The amount of noodle soup intake was determined by the question, “How much soup do you have from ramen, udon, and soba noodles?” with answers on five levels (almost never, one-third, half, two-thirds, and almost all). Here, they were divided into two groups; “≥50%” or “<50%.”

#### 2.2.3 Statistical analyses

Chi-square tests were used to compare categorical values. Cox proportional hazards analysis was performed with and without adjustment for background factors to examine the association between intake frequency and mortality. The adjusted factors included age, sex, smoking, alcohol consumption, amount of noodle soup consumed, diabetes, hypertension, and dyslipidaemia. The reference group was set as “frequency of noodle intake 1–2 times/week,” which showed the lowest mortality risk in the adjusted analysis. Additionally, subgroup analyses were performed according to sex, age, and the amount of noodle soup consumed. Since subgroup analyses in this study did not adjust for the multiplicity of p-values, the results are interpreted as exploratory. P-values are presented for reference only. The statistical software JMP pro16 for Windows was used for all statistical analyses. Statistical significance was set at P < 0.05.

#### 2.2.4 Ethical considerations

Written informed consent was obtained upon completion of the questionnaire. To collect mortality information during the follow-up period, the study outline was posted on the Yamagata University School of Medicine website and an opt-out method was used, whereby participation in the study could be withdrawn upon request from the individual. This study was approved by the Ethics Committee of Yamagata University School of Medicine (2009–1222, 2023–294) and conducted in accordance with the Declaration of Helsinki.

## 3 Results

### 3.1 Characteristics of participants (Table 1)

The participants were 6,725 individuals (34.9% men) with a mean age of 59.7 ± 6.7 years. The prevalence of ramen intake frequency was 18.9%, 46.7%, 27.0%, and 7.4% for < 1/month, 1–3 times/month, 1–2 times/week, and ≥3 times/week. The higher the frequency of ramen intake, the higher the BMI and prevalence of men, young age, smoking, alcohol consumption, ≥50% of the noodle soup consumed, diabetes, and hypertension.

**Table 1:** The characteristics of study subjects.

Frequency of ramen intake		Total	<1 /month	1−3 times/month	1−2 times/week	≥3 times/week	p-value
Number (%)		6725 (100%)	1274 (18.9%)	3149 (46.7%)	1813 (27.0%)	499 (7.4%)	
Sex	Male	2349 (34.9%)	28.3%	30.1%	43.5%	51.5%	<0.001
	Female	4376 (65.1%)	71.7%	69.9%	56.5%	48.5%	
Age	40−49	225 (3.4%)	2.3%	3.7%	3.1%	4.6%	<0.001
	50−59	844 (12.5%)	11.5%	12.7%	13.1%	12.6%	
	60−69	3020 (44.9%)	40.0%	46.5%	45.1%	46.5%	
	70−74	2636(39.2%)	46.2%	37.1%	38.7%	36.3%	
BMI (kg/m^2^)	<18.5	497 (7.4%)	9.7%	7.2%	6.3%	6.4%	0.004
	18.5−24.9	4571 (68.0%)	67.3%	69.2%	67.4%	64.3%	
	25−29.9	1451 (21.6%)	20.0%	20.8%	22.7%	26.3%	
	≥30	206 (3.1%)	3.0%	2.8%	3.5%	3.0%	
Smoking	Yes	538 (8.0%)	6.0%	6.7%	9.2%	16.8%	<0.001
	No	6187 (92.0%)	94.0%	93.3%	90.8%	83.2%	
Alcohol consumption	Yes	3038 (45.2%)	34.8%	43.1%	52.9%	56.3%	<0.001
	No	3687 (54.8%)	65.2%	56.9%	47.1%	43.7%	
Amount of noodle soup consumed	<1/2	3750 (55.8%)	66.3%	57.8%	48.5%	42.3%	<0.001
	≥1/2	2975(44.2%)	33.7%	42.2%	51.5%	57.7%	
Diabetes mellitus	Yes	764 (11.4%)	11.3%	10.2%	12.7%	13.6%	0.020
	No	5961 (88.6%)	88.7%	89.8%	87.3%	86.4%	
Hypertension	Yes	3375 (50.2%)	48.2%	48.2%	53.6%	55.1%	<0.001
	No	3350 (49.8%)	51.8%	51.8%	46.4%	44.9%	
Dyslipidemia	Yes	3697 (55.0%)	53.3%	54.8%	56.4%	55.3%	0.381
	No	3028 (45.0%)	46.7%	45.2%	43.6%	44.7%	

BMI: body mass index.

### 3.2 Association between frequency of ramen intake and mortality

During the follow-up period (median 4.5 years), there were 145 deaths (85 men and 60 women), including 100 from cancer and 29 from cardiovascular disease. Cox proportional hazards analysis was performed to examine the association between intake frequency and mortality in the overall population. In the unadjusted analysis, the hazard ratio was significantly higher trend for the “≥3 times/week” group (hazard ratio [HR]; 1.69, 95% confidence interval [CI]; 0.94–3.03) compared to the “1–2 times/week” group. Multivariate analysis adjusted for age, sex, smoking, alcohol consumption, amount of noodle soup consumed, diabetes, hypertension, and dyslipidaemia, the “≥3 times/week” group showed a non-significantly increased risk for mortality (HR; 1.52, 95% CI; 0.84–2.75), compared to the “1–2 times/week” group (Table 2). The interactions between the frequency of ramen intake and each background factor were not statistically significant.

**Table 2:** Frequency of Ramen Consumption Associated with Total Mortality.

		Unadjusted analysis		Adjusted analyses*	
Frequency of ramen intake	Number of events/subjects	HR (95%CI)	p-value	HR (95%CI)	p-value
<1 /month	145/6725	1.24 (0.77–2.00)	0.384	1.43 (0.43–1.15)	0.157
1−3 times/month	32/1275	0.92 (0.61–1.39)	0.692	1.09 (0.71–1.66)	0.705
1−2 times/week	59/3139	reference		reference	
≥3 times/week	17/499	1.69 (0.94–3.03)	0.076	1.52 (0.84–2.75)	0.163

HR: hazard ratio. CI: confidence interval.

* Multivariate analysis adjusted for age, sex, smoking, alcohol consumption, amount of noodle soup consumed, diabetes, hypertension, dyslipidemia.

### 3.3 Subgroup analysis: association between frequency of ramen noodle intake and total mortality

Multivariate analyses of the subgroups stratified by background factors were performed (Table 3). In men, the HR for mortality was significantly higher in the “<1/month” (HR; 2.07, 95% CI; 1.09–3.97) groups, compared to the “1–2 times/week” group. In participants <70 years of age, the HR for mortality associated with ramen consumption less than once/month was (HR; 2.17, 95% CI; 1.08–4.34) compared to “1–2 times/week” and the HR for mortality for “≥3 times/week” was significantly increased (HR; 2.20, 95% CI; 1.03–4.73). In participants with ≥50% of noodle soup consumed, the HR for mortality was significantly higher in the “<1/month” (HR; 2.43, 95% CI; 1.20–4.92) groups, but not in those with <50% of noodle soup consumed. In terms of alcohol consumption, the hazard ratio (HR) for mortality was significantly higher in the “≥3 times/week” group (HR; 2.71, 95%CI; 1.33–5.56). Additionally, the comparison was made on the basis of less than once a month. The results were similar to those in Table 2, which was based on 1-2 times per week. (Supplementary file)

**Table 3:** Association between frequency of ramen intake and total mortality (subgroup analyses).

Frequency of ramen intake	Number of events/subjects	Unadjusted analysis		Adjusted analyses*	
		HR (95%CI)	p-value	HR (95%CI)	p-value
Male	85/2349				
<1 /month	19/360	2.04 (1.08–3.83)	0.028	2.07 (1.09–3.97)	0.027
1−3 times/month	33/944	1.32 (0.76–2.31)	0.323	1.40 (0.80–2.46)	0.240
1−2 times/week	21/788	reference		reference	
≥3 times/week	12/257	1.79 (0.87–3.69)	0.115	1.74 (0.83–3.65)	0.140
Female	60/4376				
<1 /month	13/914	0.91 (0.44–1.90)	0.802	0.85 (0.40–1.80)	0.672
1−3 times/month	26/2195	0.76 (0.40–1.42)	0.382	0.74 (0.39–1.39)	0.301
1−2 times/week	16/1025	reference		reference	
≥3 times/week	5/242	1.33 (0.48–3.67)	0.581	1.23 (0.44–3.43)	0.685
Age <70	75/4089				
<1 /month	18/686	1.73 (0.89–3.39)	0.107	2.17 (1.08–4.34)	0.029
1−3 times/month	28/1974	0.93 (0.53–1.65)	0.809	1.07 (0.58–2.00)	0.821
1−2 times/week	17/1111	reference		reference	
≥3 times/week	12/318	2.52 (1.19–5.34)	0.016	2.20 (1.03–4.73)	0.043
Age ≥70	70/2636				
<1 /month	14/588	0.83 (0.42–1.66)	0.602	0.95 (0.47–1.94)	0.898
1−3 times/month	31/1165	0.93 (0.53–1.65)	0.809	1.11 (0.62–1.99)	0.730
1−2 times/week	20/702	reference		reference	
≥3 times/week	5/181	0.97 (0.36–2.62)	0.950	0.91 (0.33–2.48)	0.850
Noodle soup <1/2	72/3750				
<1 /month	16/845	0.87 (0.45–1.71)	0.696	0.91 (0.46–1.79)	0.776
1−3 times/month	31/1814	0.78 (0.44–1.40)	0.418	0.90 (0.50–1.62)	0.733
1−2 times/week	19/880	reference		reference	
≥3 times/week	6/211	1.32 (0.52–3.36)	0.552	1.25 (0.31–2.05)	0.640
Noodle soup ≥1/2	73/2975				
<1 /month	16/429	1.96 (0.99–3.90)	0.052	2.43 (1.20–4.92)	0.014
1−3 times/month	28/1325	1.10 (0.60–2.00)	0.761	1.27 (0.69–2.33)	0.443
1−2 times/week	18/933	reference		reference	
≥3 times/week	11/288	2.02 (0.94–4.33)	0.071	1.76 (0.81–3.85)	0.153
Alcohol (+)	71/3038				
<1 /month	13/443	1.58 (0.77–3.26)	0.213	1.76 (0.84–3.69)	0.132
1−3 times/month	25/1354	0.98 (0.53–1.81)	0.960	1.12 (0.60–2.09)	0.713
1−2 times/week	18/960	reference		reference	
≥3 times/week	15/281	2.95 (1.47–5.93)	0.002	2.71 (1.33–5.56)	0.006
Alcohol (−)	74/3687				
<1 /month	19/831	1.03 (0.53–1.95)	0.935	1.16 (0.60–2.25)	0.654
1−3 times/month	34/1785	0.85 (0.48–1.50)	0.581	1.02 (0.57–1.83)	0.936
1−2 times/week	19/853	reference		reference	
≥3 times/week	2/218	0.41 (0.09–1.76)	0.228	0.36 (0.08–1.57)	0.174

HR: hazard ratio. CI: confidence interval.

* Multivariate analysis adjusted for age, sex, smoking, alcohol consumption, amount of noodle soup consumed, diabetes, hypertension, dyslipidemia.

## 4 Discussion

This study showed that Japanese community residents consume ramen daily and frequent intake is associated with high BMI, smoking, alcohol consumption, diabetes, hypertension, and consumption of noodle soup. Furthermore, frequent ramen intake was associated with increased mortality risk in several groups, including men, those aged <70 years, and those who consumed ≥50% of noodle soup and alcohol.

### 4.1 Frequency of ramen intake and background factors in the general population

Here, the frequency of noodle intake was 1–3 times/month (46.7%), followed by 1–2 times/week (27.0%), indicating that most of the Japanese population consumed noodles daily. Those who frequently consumed ramen were more likely to be men, younger, and have a higher BMI, smoking, alcohol consumption, ≥50% of noodle soup consumed, diabetes, and hypertension. These study results align with those of a previous study in which noodles were often consumed by men [9] and participants with alcohol consumption [10]. Frequent ramen intake is accompanied by increased noodle soup consumption, which results in excessive sodium intake [3]. Accordingly, participants who consume ramen frequently are at a higher risk of developing cardiovascular diseases and cancer [11], which are associated with mortality.

### 4.2 Association between the frequency of ramen intake and mortality

Excessive salt intake leads to the development of cardiovascular diseases, such as stroke [12,13] and gastric cancer [14]. Ramen generally contains a large amount of salt [3]; therefore, their frequent intake may be associated with mortality via the development of these diseases. Presently, the association between ramen intake frequency and mortality was not significant in the total population. However, it was significant in several subgroups, such as men, those aged <70 years, and those who consumed more noodle soup and alcohol. This indicates that background factors of the participants may have modulated the association between ramen intake and mortality.

Presently, the association between frequency of ramen intake and mortality was significant in young adults and men. Young individuals and men tended to consume larger amounts of ramen per serving (large servings), in which salt intake would increase. It has been reported that a high percentage of sodium intake in young men comes from noodles [3], suggesting that the higher frequency of ramen intake among young men is more strongly correlated with a higher daily sodium intake. Moreover, the increased risk of gastric cancer due to excessive salt intake is more pronounced in men. These findings strengthen the association between intake frequency and mortality in these groups. Interestingly, the mortality risk in participants with ≥3 times/week ramen intake was higher in those who consumed ≥50% of noodle soup and alcohol. This indicates that a large amount of soup and alcohol consumed may strengthen the association between dietary intake and mortality.

The mortality risk of the group that consumed the least amount of ramen (<1/month) was higher in men and those who consumed ≥50% of noodle soup. The underlying mechanism is unknown; however, participants with hypertension, diabetes, or cardiovascular comorbidities may be instructed to reduce their ramen intake. The frailty might be an important issue, especially in older age groups and would like to consider this as future research.

The strengths of this study include the relatively large sample size and various subgroup analyses. Knowingly, this is the first study to examine the association between ramen intake frequency and total mortality in the general population. However, this study has several limitations. First, a direct causal relationship cannot be determined because this was an observational study, and this study uses cross-sectional dietary data, and temporal changes in diet are not considered. Second, information on factors, such as the amount of ramen consumed per serving (g), intake of other foods, and exercise habits was unavailable. The number of events by cause of death was insufficient to allow for an analysis by cause of death. The validity/reliability of food frequency questionnaires has been examined by Imaeda et al., and it is said that they can be used for epidemiological surveys based on the results of correlation coefficients [15]. However, the questionnaire relies on self-reported data and broadly defined categories, lacking details on portion size and type of ramen consumed. This may result in discrepancies between reported and actual intake. Third, the results of the subgroup analyses are presented for reference purposes without correction for multiple comparisons. Further investigation is warranted. Due to the lack of data on factors such as total energy intake, socioeconomic status, burden of comorbidities (e.g., CKD, history of cancer), and physical activity, these variables could not be adjusted for in our analysis. This limitation may result in residual confounding. Future studies should incorporate these factors in data collection and analysis.

## 5 Conclusions

This study shows that Japanese community residents with various comorbidities frequently consume ramen noodles. Furthermore, frequent ramen intake was associated with mortality risk in men, those aged <70 years, and those who consumed ≥50% of noodle soup and alcohol. These results suggest that the appropriate intake of ramen noodles may be recommended based on individual characteristics.

## Statements and declarations

The questionnaire package contained an overview of the study, including information on the voluntary nature of participation and protection of personal information. Written informed consent was obtained from all the participants. This study was conducted in accordance with the Declaration of Helsinki and approved by the Ethics Committee of Yamagata University School of Medicine (2009−1222, 2023−294).

## Funding

The Yamagata Cohort Study is supported by the 21st Century COE Program and Global COE Program.

## Declaration of competing interest

The authors declare no competing interests.
