# Corrigendum to “Frequent Ramen consumption and increased mortality risk in specific subgroups: A Yamagata cohort study” [J Nutr Health Aging, 29 (2025) 100643]

**DOI:** 10.1016/j.jnha.2025.100684

**Published:** 2025-09-20

**Authors:** Miho Suzuki, Natsuko Suzuki, Ri Sho, Masayoshi Souri, Tsuneo Konta

**Affiliations:** a Department of Health and Nutrition, Yamagata Prefectural Yonezawa University of Nutrition Sciences, Japan; b Department of Public Health and Hygiene, Yamagata University School of Medical Science, Japan

The authors recently identified an error in Table 2: Adjusted analyses for <1/month should be 1.43 (0.43–2.15).

The authors would like to apologise for any inconvenience caused.

