# Supplementary material for: Frequent Ramen consumption and increased mortality risk in specific subgroups: A Yamagata cohort study
Source: J Nutr Health Aging. 2025 Aug 1;29(10):100643. doi: 10.1016/j.jnha.2025.100643 (PMC12337647; doi:10.1016/j.jnha.2025.100643)
Supplement: Supplementary file 1 [file mmc1.pdf]

Supplementary File. Association between frequency of ramen intake and total mortality

Frequency of ramen intake	Number of events/subjects	Unadjusted analysis		Adjusted analyses*	
		HR (95%CI)	p-value	HR (95%CI)	p-value
Total	145/6725				
<1 /month	32/1274	reference		reference	
1-3 times/month	59/3139	0.74 (0.48–1.15)	0.182	0.76 (0.49–1.18)	0.225
1-2 times/week	37/1813	0.81 (0.50–1.31)	0.384	0.70 (0.43–1.15)	0.157
≥3 times/week	17/499	1.69 (0.95–3.03)	0.077	1.52 (0.84–2.75)	0.163
Male	85/2349				
<1 /month	19/360	reference		reference	
1-3 times/month	33/944	0.65 (0.36–1.16)	0.144	0.67 (0.37–1.22)	0.192
1-2 times/week	21/788	0.49 (0.26–0.93)	0.028	0.48 (0.25–0.92)	0.027
≥3 times/week	12/257	0.88 (0.42–1.84)	0.733	0.84 (0.39–1.81)	0.657
Female	60/4376				
<1 /month	13/914	reference		reference	
1-3 times/month	26/2195	0.83 (0.43–1.62)	0.588	0.87 (0.44–1.70)	0.680
1-2 times/week	16/1025	1.09 (0.53–2.30)	0.801	1.18 (0.56–2.48)	0.672
≥3 times/week	5/242	1.46 (0.52–4.14)	0.475	1.23 (0.44–3.43)	0.685
Age <70	75/4089				
<1 /month	18/686	reference		reference	
1-3 times/month	28/1974	0.53 (0.29–0.97)	0.040	0.50 (0.27–0.91)	0.024
1-2 times/week	17/1111	0.58 (0.30–1.13)	0.107	0.46 (0.23–0.92)	0.029
≥3 times/week	12/318	1.46 (0.69–3.06)	0.322	1.02 (0.46–2.22)	0.968
Age ≥70	70/2636				
<1 /month	14/588	reference		reference	
1-3 times/month	31/1165	1.12 (0.59–2.12)	0.727	1.16 (0.61–2.22)	0.651
1-2 times/week	20/702	1.20 (0.60–2.40)	0.602	1.05 (0.51–2.13)	0.898
≥3 times/week	5/181	1.16 (0.41–3.28)	0.773	0.95 (0.33–2.73)	0.925
Noodle soup <1/2	72/3750				
<1 /month	16/845	reference		reference	
1-3 times/month	31/1814	0.90 (0.49–1.66)	0.737	0.99 (0.53–1.84)	0.993
1-2 times/week	19/880	1.14 (0.58–2.24)	0.696	1.10 (0.55–2.18)	0.776
≥3 times/week	6/211	1.52 (0.59–3.92)	0.391	1.38 (0.53–3.62)	0.510
Noodle soup ≥1/2	73/2975				
<1 /month	16/429	reference		reference	
1-3 times/month	28/1325	0.56 (0.30–1.04)	0.066	0.52 (0.28–0.99)	0.047
1-2 times/week	18/933	0.51 (0.26–1.01)	0.052	0.41 (0.20–0.83)	0.004
≥3 times/week	11/288	1.03 (0.47–2.24)	0.951	0.73 (0.32–1.65)	0.445
Alcohol (+)	71/3038				
<1 /month	13/443	reference		reference	
1-3 times/month	25/1354	0.90 (0.49–1.66)	0.737	0.99 (0.53–1.84)	0.993
1-2 times/week	18/960	1.14 (0.58–2.24)	0.696	1.10 (0.55–2.18)	0.776
≥3 times/week	15/281	1.52 (0.59–3.92)	0.391	1.38 (0.53–3.62)	0.510
Alcohol (-)	74/3687				
<1 /month	19/831	reference		reference	
1-3 times/month	34/1785	0.56 (0.30–1.04)	0.066	0.52 (0.28–0.99)	0.047
1-2 times/week	19/853	0.51 (0.26–1.01)	0.052	0.41 (0.20–0.83)	0.004
≥3 times/week	2/218	1.03 (0.47–2.24)	0.951	0.73 (0.32–1.65)	0.445

HR: hazard ratio. CI: confidence interval.

*Multivariate analysis adjusted for age, sex, smoking, alcohol consumption, amount of noodle soup consumed, diabetes, hypertension, dyslipidemia
